# Emotionen bei chronifiziertem Schmerz: Veränderungen im Verlauf einer tagesklinischen interdisziplinären multimodalen Schmerztherapie

**DOI:** 10.1007/s00482-023-00748-z

**Published:** 2023-08-29

**Authors:** Anne Juliane Körner, Rainer Sabatowski, Ulrike Kaiser

**Affiliations:** 1 UniversitätsSchmerzCentrum (USC), Universitätsklinikum Carl Gustav Carus Dresden, Fetscherstraße 74, 01307 Dresden, Deutschland; 2 Klinik für Anästhesiologie und Intensivmedizin, Universitätsklinikum Schleswig-Holstein, Ratzeburger Allee 160, 23538 Lübeck, Deutschland

**Keywords:** Emotionales Erleben, Emotionale Kompetenz, Primärer chronifizierter Schmerz, Interdisziplinäre multimodale Schmerztherapie, Verlaufsmessung, Emotional experience, Emotional competence, Primary chronic pain, Interdisciplinary multimodal pain therapy, Follow-up assessment

## Abstract

**Fragestellung:**

In der vorliegenden Studie wurde untersucht, inwiefern sich das emotionale Erleben und die emotionale Kompetenz (EK) bei Menschen mit chronifizierten Schmerzen während einer interdisziplinären multimodalen Schmerztherapie (IMST) verändern.

**Methoden:**

Die Untersuchung fand an *N* = 184 erwachsenen deutschsprachigen Personen mit nichttumorbedingten chronifizierten Schmerzen statt. Sie absolvierten eine tagesklinische IMST. Die Häufigkeit spezifischer Emotionen und die EK wurden zu drei Messzeitpunkten mittels des Fragebogens zur emotionsspezifischen Selbsteinschätzung emotionaler Kompetenzen (SEK-ES) und des Emotionale-Kompetenz-Fragebogens (EKF) erfasst. Die Verlaufsergebnisse wurden deskriptiv, inferenzstatistisch und mittels linearer Regression ausgewertet.

**Ergebnisse:**

Positive Emotionen wurden nach der Therapie häufiger (Effektstärke *r* = 0,40; *p* < 0,001) und negative Emotionen seltener (*r* = 0,39*; p* < 0,001) erlebt. Das Erleben von Ärger verringerte sich besonders stark (*r* = 0,52; *p* < 0,001). Die selbst eingeschätzte EK änderte sich *nicht* während der IMST (*χ*^2^_EKF__*_gesamt*_ (2) = 0,09; *p* *=* 0,956). EK erklärt in großem Ausmaß die Varianz des Häufigkeitserlebens positiver (*R*^2^ = 0,468) und negativer Emotionen (*R*^2^ = 0,390).

**Diskussion:**

Es konnten Verbesserungen der von den Patient*innen berichteten *Häufigkeiten positiver und negativer Emotionen* während der IMST nachgewiesen werden. Weiterführende Forschung sollte die Ergebnisse mittels Kontrollgruppe validieren. Auch wenn für die untersuchten Personen kein expliziter Kompetenzzuwachs wahrnehmbar war, hatte die EK einen hohen prädiktiven Wert für die Emotionshäufigkeit. Zukünftige Therapiekonzeptionen und -evaluierungen sollten die Veränderungen des emotionalen Erlebens stärker fokussieren.

**Graphic abstract:**

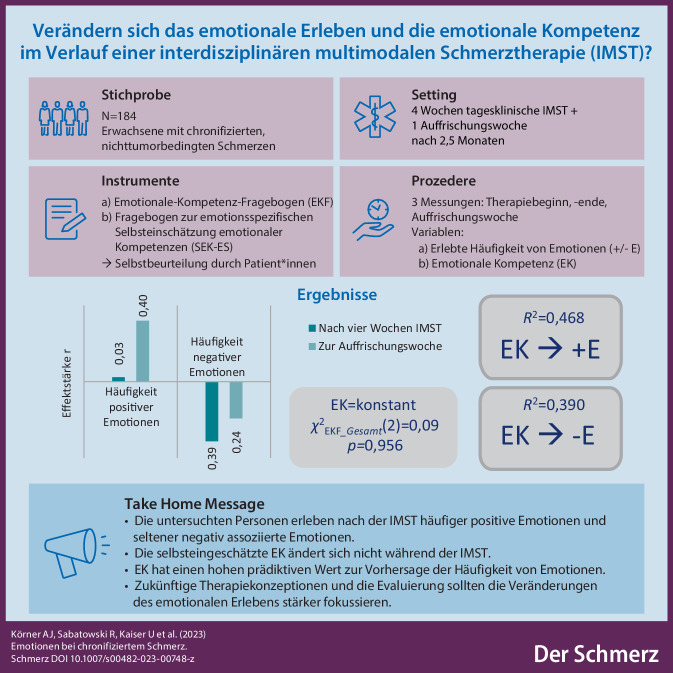

## Abstract (en)

**Research question:**

The present study examined the extent to which emotional experience and emotional competence (EC) change in people with chronic pain during interdisciplinary multimodal pain treatment (IMPT).

**Methods:**

The study included *N* = 184 adult German-speaking individuals with non-cancer-related chronic pain. They completed a day clinic IMPT. The frequency of specific emotions (anger, etc.) and EC was assessed at three measurement time points using the Questionnaire for Emotion-Specific Self-Assessment of Emotional Competencies (ERSQ-ES) and the Emotional Competency Questionnaire (ECQ). The course results were analyzed descriptively, inferentially, and using linear regression.

**Results:**

Positive emotions were experienced more frequently (effect size *r* = 0.40; *p* < 0.001) and negative emotions less frequently (*r* = 0.39, *p* < 0.001) at end of therapy. The experience of anger decreased particularly strongly (*r* = 0.52; *p* < 0.001). Self-assessed EC did not change during the IMPT (*χ*^2^_ECQ_total_ (2) = 0.09; *p* = 0.956). EC largely explained the variance in the frequency experience of positive (*R*^2^ = 0.468) and negative emotions (*R*^2^ = 0.390).

**Discussion:**

Improvements in patient-reported *frequencies of positive and negative emotions* during IMPT were demonstrated. Further research should validate these results using a control group. Even though no explicit increase in competence was perceivable for the studied subjects, EC had a high predictive value for emotion frequency. Future therapy designs and evaluations should focus more on changes of emotional experience.

## Einleitung

### Was ist eine Emotion?

„Emotionen werden in zielrelevanten Situationen ausgelöst und signalisieren, dass etwas unserer Aufmerksamkeit bedarf. Außerdem beinhalten sie eine subjektive, physiologische und Verhaltenskomponente. Typische Charakteristika sind Instabilität, Intensität, die kurze Dauer und Gerichtetheit“ [1]. In Abgrenzung zu dieser von Barnow zusammenfassenden Definition des Konzepts „Emotion“ lassen sich Affekte als niederschwellige interozeptive sensorische Signalgebung mit Empfindungen von Annehmlichkeit/Unannehmlichkeit und Aktivierung/Deaktivierung einordnen [2]. Erst nach Abgleich mit dem eigenen konzeptuellen Wissen, welches auf der bisherigen kulturellen und biografischen Erfahrung basiert, erhält der Affekt eine Bedeutung [3]. Affekt und Bedeutung bilden letztlich das emotionale Erleben. Emotionen können weiterhin von Stimmungen abgegrenzt werden. Hierbei handelt es sich um diffuse und unfokussierte Zustände, die, im Gegensatz zu Emotionen, langfristiger sind und sich langsam verändern [4].

### Was ist emotionale Kompetenz?

Emotionale Kompetenz (EK) umfasst „die Fähigkeit zum Erkennen und Ausdrücken von Emotionen sowie zu einem angemessenen Umgang mit Gefühlen“ [5]. Vor dem Hintergrund dieser Definition kann emotionale Kompetenz als die Fertigkeit einer Person verstanden werden, Emotionen bei sich selbst bzw. anderen zu erkennen, eigene Emotionen zu regulieren und über eine adaptive emotionale Expressivität zu verfügen [5]. Berking (2015) differenziert folgende Subkompetenzen: die Kompetenz, die eigenen Emotionen bewusst wahrzunehmen, sie klar zu erkennen/benennen, die Ursachen der aktuell erlebten Emotion zu verstehen, sich selbst in belastenden Situationen effektiv zu unterstützen, das emotionale Erleben gezielt positiv verändern zu können, negativ erlebte Emotionen zu akzeptieren, gegebenenfalls auszuhalten und sich emotional belastenden Situationen auszusetzen, um persönliche Ziele zu erreichen [6].

### Emotionen und chronifizierter Schmerz

Das emotionale Erleben ist Teil der Schmerzdefinition [7]. Negativ erlebte Emotionen sind mit Schmerzerleben assoziiert [8]. Vor dem Hintergrund überlappender neuronaler Aktivierungsmuster im Gehirn [8, 9] und emotionsabhängiger muskulärer Anspannung im Körper [10] ist diese Kopplung erklärbar. Biopsychosoziale Betrachtungen lang andauernder/wiederkehrender und beeinträchtigender Schmerzen führten zu der Erkenntnis, dass Emotionen einen wesentlichen Bestandteil in der Konzeptualisierung, Bewertung und Behandlung chronifizierter Schmerzen darstellen [11]. Dabei sind insbesondere breiter aufgestellte Behandlungen zur Verarbeitung von Emotionen, beispielsweise „eye movement desensitization and reprocessing“ (EMDR; [12]) und „emotional awareness and expression therapy“ (EAET; [13, 14]), vielversprechende Optionen zur Reduktion chronischer Schmerzen [15].

### Veränderung von Emotionen und emotionalen Kompetenzen während der Schmerztherapie

In der Literatur finden sich bisher wenige Untersuchungen, die konkret die emotionalen Veränderungen während einer interdisziplinären multimodalen Schmerztherapie (IMST) erfassen. Das Verständnis der IMST beruht hierbei auf der durch die Ad-hoc-Kommission bestimmten Definition [16].

Einige Studien konnten eine Reduktion von *Angst* und der im weiteren Sinne emotionsassoziierten *Depressivität* während einer tagesklinischen IMST nachweisen [17, 18]. Genutzt wurden hierzu die Fragebögen, die zum damaligen Zeitpunkt im deutschen Schmerzfragebogen verankert waren: die Allgemeine Depressionsskala und die Hospital Anxiety and Depression Scale [19]. Pardos-Gascón et al. [20] konzentrierten ihre Überblicksarbeit auf rein achtsamkeitsbasierte oder kognitiv-behaviorale Interventionsstudien und extrahierten die Reduktion von *Angst, Depressivität* und dem emotionsassoziierten *Stresserleben* als häufiges Behandlungsergebnis.

Studien, die die potenziellen Veränderungen konkreter positiv und negativ erlebter Emotionen, z. B. Ärger oder positiver Bewältigungsemotionen, während einer IMST in den Mittelpunkt stellen und mit emotionsspezifischen Instrumenten erheben, finden sich kaum. *Bewältigungsemotionen* umfassen hierbei Emotionen wie Dankbarkeit, Mut und Zuversicht [21] und dienen der konstruktiven Bewältigung alltäglicher und nicht alltäglicher Herausforderungen.

Auch Verlaufsmessungen der mit den Emotionen in Verbindung stehenden emotionalen Kompetenz fehlen bisher.

Die vorliegende Untersuchung möchte nun genau auf diesen Punkt fokussieren: Verändern sich das emotionale Erleben und die Häufigkeit bestimmter Emotionen bei Menschen mit chronifizierten Schmerzen während einer tagesklinischen IMST? Und verändert sich die wahrgenommene emotionale Kompetenz während der IMST?

## Material und Methoden

### Stichprobe

Es wurden 184 Patient*innen einer tagesklinischen interdisziplinären multimodalen Schmerztherapie (IMST) des Universitätsschmerzzentrums Dresden inkludiert (siehe Stichprobe in Körner et al. [22]). Es handelte sich dabei um erwachsene Menschen mit chronifizierten, nichttumorbedingten Schmerzen. Die Auswahl fand anhand IMST-spezifischer Einschlusskriterien statt [16, 23].

### Setting

Das Behandlungskonzept der Schmerztagesklinik und der Gruppenpsychotherapie im Allgemeinen kann bei Schütze et al. [17] nachvollzogen werden. Im Speziellen sei an dieser Stelle der emotionsfokussierte Anteil der Schmerzpsychotherapie umrissen. Durch tägliche Selbstbeobachtungsübungen in den Gruppenpsychotherapien lag die Aufmerksamkeit unter anderem auf dem emotionalen Erleben und den dahinterliegenden Bedürfnissen. Emotionsfokussierte Fragetechniken unterstützten die Exploration. Psychoedukativ wurden die Teilnehmenden der Gruppenpsychotherapie angeleitet, den Einfluss negativ erlebter Emotionen in ein Gesamtschmerzmodell einzuordnen. Der Einsatz von bildhafter Sprache, Bildimpulsen, Geschichten und Metaphern diente der Annäherung an emotionale Inhalte. Die Anwendung imaginativer Techniken verhalf zur Stimulation relevanter Emotionen. Ziel der Aktivierung bedeutsamer Emotionen war, diese annehmen zu lernen, die dahinterstehenden Bedürfnisse zu verstehen und einen adaptiven Umgang zu entwickeln.

### Instrumente

A. *Fragebogen zur emotionsspezifischen Selbsteinschätzung emotionaler Kompetenzen (SEK-ES)*
  Die Häufigkeit bestimmter Emotionen und die emotionale Kompetenz wurden mit dem Fragebogen zur emotionsspezifischen Selbsteinschätzung emotionaler Kompetenzen (SEK-ES [21]) ermittelt. Die konzeptuelle Ausgangsbasis des Fragebogens bildete das kompetenzorientierte Modell [6]. In Teil A des SEK-ES wurde zunächst das Auftreten diverser Emotionen und Stimmungen innerhalb der vergangenen Woche erfasst. Neben Stress, Angst, Ärger, Traurigkeit, Depressivität, Schuld, Scham und Ekel wurde außerdem die Häufigkeit von Bewältigungsemotionen erfragt. Laut Auswertungsalgorithmus [21] handelt es sich bei den Bewältigungsemotionen um eine Subkategorie der positiv erlebten Emotionen. Bewältigungsemotionen umfassen Emotionen wie Dankbarkeit, Mut, Zuversicht oder Stolz. Die übergeordnete Kategorie der positiven Emotionen beinhaltet zusätzlich zu den Bewältigungsemotionen noch Emotionen wie Zufriedenheit, Freude oder Liebe. Schließlich wurden in Teil B einzelne emotionale Reaktionen mit je zwölf Items genauer untersucht, falls die jeweilige emotionale Reaktion in der letzten Woche auftrat. Folgende emotionale Qualitäten wurden dabei erfragt: *Stress/Anspannung, Angst, Ärger, Traurigkeit, depressive Stimmung, weitere belastende Gefühle, positive Gefühle *(SEK-ES [21]). Die Häufigkeiten der Emotionen (Teil A) und der konstruktive Umgang mit spezifischen Emotionen (Teil B) wurden anhand eines durch die Testautor*innen festgelegten Algorithmus berechnet [21]. Es handelte sich hierbei um Mittelwerte.
B. *Emotionale-Kompetenz-Fragebogen als Selbstbeurteilungsversion (EKF)*
  Die *emotionale Kompetenz* wurde weiterhin mittels der Selbstbeurteilungsskalen des Emotionale-Kompetenz-Fragebogens (EKF [5]) erfasst. Die Bearbeitungszeit des EKF dauerte zehn bis zwanzig Minuten. Der Fragebogen bestand aus vier Hauptskalen: *Erkennen eigener Emotionen, Erkennen von Emotionen bei anderen, Regulation und Kontrolle eigener Emotionen und emotionale Expressivität.* Für den Selbsteinschätzungsfragebogen lag eine vom Autor zur Verfügung gestellte Normstichprobe (*N* = 638) vor [5].

Die Besonderheit des SEK-ES lag in der *emotionsspezifischen* Aufschlüsselung von Häufigkeiten und konstruktivem Umgang. Der EKF hingegen ermöglichte durch die vom Autor vorgegebenen *Normwerte* einen Abgleich der Ergebnisse mit der Normalbevölkerung (verkörpert durch die Normstichprobe; [5]).

### Prozedere

Die Datenerfassung erfolgte zu drei Messzeitpunkten: zu Beginn der tagesklinischen Behandlung (erste Woche, T1), am Ende der vierwöchigen tagesklinischen Behandlung (T2) und zur Auffrischungswoche (14 Wochen nach Therapiebeginn, T3). Die Selbstbeurteilungsinstrumente wurden der standardmäßigen Verlaufsbeurteilung der Schmerztherapie mittels des Deutschen Schmerzfragebogens [19] beigefügt. Von den zu Therapiebeginn ursprünglich 209 rekrutierten Patient*innen waren die Daten für 184 Patient*innen zu allen drei Messzeitpunkten vollständig. Die Datenerfassung erfolgte von Januar 2018 bis Juni 2019.

### Statistische Auswertung

Die Rohwerte wurden durch den Abgleich mit der Normstichprobe des EKF in Standardnormwerte umgewandelt. Für jede untersuchte Person konnte anhand des Standardnormwerts das Ausmaß der emotionalen Kompetenz im Verhältnis zur Normalbevölkerung festgestellt werden (90–110 durchschnittlich, < 90 unterdurchschnittlich, > 110 überdurchschnittlich [5]).

Für die Daten des SEK-ES und des EKF wurden Mittelwerte und Standardabweichungen für alle drei Messzeitpunkte erfasst. Nach Überprüfung der statistischen Voraussetzungen erfolgte der inferenzstatistische Vergleich intraindividueller Ergebnisse über die drei Messzeitpunkte hinweg mittels des nonparametrischen Friedman-Tests. Effekte wurden mittels der Effektstärke *r *quantifiziert.

Zum verbesserten Verständnis der Einflussstärke der emotionalen Kompetenz (Prädiktor) auf die Häufigkeit positiver und negativer Emotionen (Kriterium) zum Therapieende (T3) wurde retrospektiv eine einfache lineare Regression durchgeführt. Da bei der Variable *Häufigkeit negativer Emotionen* im Breusch-Pagan-Test Heteroskedastizität nachgewiesen wurde, erfolgte eine zusätzliche Absicherung des Regressionsmodells mittels Bootstrapping. Zur Bemessung der Effektgröße diente der Determinationskoeffizient *R*^2^.

## Ergebnisse

### Veränderung der Häufigkeiten von Emotionen

Bezüglich der *erlebten Häufigkeit positiver und negativer Emotionen* fielen deutliche Veränderungen auf. So berichteten die untersuchten Personen zu T3 entscheidend häufiger positive Emotionen als zu T1 (T1/T3: *z* = −5,28; *p* < 0,001; *r* = 0,40, Tab. 1 und 2). Zudem wurde deutlich, dass die untersuchten Personen statistisch signifikant weniger *negative Emotionen *im Verlauf schilderten (Tab. 1 und 2). Im Gegensatz zu den positiven Emotionen, bei denen die größte Veränderung zu T3 erreicht war, zeigten sich bei der *erlebten Häufigkeit negativer Emotionen* bereits zu T2 die größten Effekte (T2/T1: z = 5,17; *p* < 0,001; *r* = 0,39, Tab. 1 und 2).

**Tab. 1:** Deskriptive Statistik der Sub- und Gesamtskalen des SEK-ES und des EKF (T1; T2; T3)

	Sub‑/Gesamtskalen	T1		T2		T3	
		*M*	*SD*	*M*	*SD*	*M*	*SD*
*SEK-ES*	*H von negativen Emotionen*	1,27	0,70	1,00	0,69	1,10	0,67
	*H von positiven Emotionen*	2,14	0,76	2,24	0,63	2,45	0,76
	H von Stresserleben	2,07	0,81	1,65	0,87	1,73	0,85
	H von Angst	1,56	0,78	1,32	0,80	1,30	0,81
	H von Ärger	1,40	0,78	0,97	0,71	1,19	0,72
	H von Traurigkeit	1,73	0,90	1,48	0,88	1,45	0,90
	H von depressiver Stimmung	1,30	0,91	1,03	0,85	1,09	0,87
	H von Scham	0,69	0,69	0,55	0,66	0,58	0,67
	H von Ekel	0,47	0,72	0,37	0,63	0,38	0,65
	H von Schuld	0,90	0,98	0,69	0,87	0,79	0,87
	H von Bewältigungsemotionen	2,22	0,76	2,47	0,70	2,47	0,77
	*KU gesamt*	2,54	0,63	2,51	0,67	2,59	0,63
	KU mit Stresserleben	2,28	0,64	2,34	0,69	2,37	0,60
	KU mit Angst	2,61	0,81	2,52	0,85	2,36	0,76
	KU mit Ärger	2,63	0,67	2,45	0,74	2,48	0,68
	KU mit Traurigkeit	2,46	0,78	2,40	0,82	2,33	0,76
	KU mit depressiver Stimmung	2,05	0,76	1,94	0,75	1,91	0,74
	KU mit positiven Emotionen	2,74	0,79	2,70	0,74	2,88	0,72
*EKF*	*Emotionale Kompetenz*	99,58	7,92	99,31	7,78	99,65	7,99
	Erkennen eigener Emotionen	99,98	10,77	99,13	10,49	99,84	10,52
	Erkennen von Emotionen bei anderen	100,50	11,05	100,32	11,28	100,07	12,16
	Regulation eigener Emotionen	100,35	9,47	100,19	9,37	100,63	9,39
	Emotionale Expressivität	97,47	10,99	97,53	11,03	98,07	10,88

Gesamtskalen sind *kursiv* hervorgehoben

*EKF* Emotionale-Kompetenz-Fragebogen, *SEK-ES* Fragebogen zur emotionsspezifischen Selbsteinschätzung emotionaler Kompetenzen, *H* erlebte Häufigkeit, *KU* konstruktiver Umgang, *T1* Therapiebeginn, *T2* Therapieende nach 4 Wochen IMST, *T3* Auffrischungswoche, 14 Wochen nach Therapiebeginn, *M* Mittelwert, *SD* Standardabweichung

**Tab. 2:** Friedman-Test und Post-hoc-Tests nach Dunn-Bonferroni zur Erfassung signifikanter Veränderung im Rahmen der Messwiederholung (T1; T2; T3) der Emotionshäufigkeit und der emotionalen Kompetenz

		*n*	*χ*^*2*^	Power in %^a^	Post-hoc-Tests nach Dunn-Bonferroni		
					Messzeitpunkte	Standardteststatistik^b^	*r*
*SEK-ES*	*H von negativen Emotionen*	174	28,78**	100	T2/T1	5,17**	0,39
					T3/T1	3,11**	0,24
					T2/T3	−2,06	0,16
	*H von positiven Emotionen*	174	35,91**	100	T1/T2	−0,35	0,03
					T1/T3	−5,28**	0,40
					T2/T3	−4,93**	0,37
	H von Stress	179	46,60**	100	T2/T1	6,00**	0,45
					T3/T1	4,45**	0,33
					T2/T3	−1,53	0,11
	H von Angst	178	23,11**	100	T2/T1	3,52*	0,26
					T3/T1	3,95**	0,30
					T3/T2	0,42	0,03
	H von Ärger	177	59,71**	100	T2/T1	6,91**	0,52
					T3/T1	3,22*	0,24
					T2/T3	−3,69*	0,28
	H von Traurigkeit	178	23,20**	100	T2/T1	3,39*	0,25
					T3/T1	4,08**	0,31
					T3/T2	0,69	0,05
	H von depressiver Stimmung	178	20,28**	100	T2/T1	3,68*	0,28
					T3/T1	3,15*	0,24
					T2/T3	−0,53	0,04
	H von Scham	177	9,96*	100	T2/T1	2,18	0,16
					T3/T1	2,29	0,17
					T3/T2	0,11	0,01
	H von Bewältigungsemotionen	174	37,99**	100	T1/T2	−4,61**	0,35
					T1/T3	−5,52**	0,42
					T2/T3	−0,91	0,07
	H von Ekel	179	4,99	100	–	–	–
	H von Schuld	179	7,09	100	–	–	–
	*KU gesamt*	158	2,63	100	–	–	–
	KU mit Stress	124	4,39	100	–	–	–
	KU mit Angst	48^c^	0,75	99,9	–	–	–
	KU mit Ärger	69^c^	1,93	99,9	–	–	–
	KU mit Traurigkeit	59^c^	0,99	99,2	–	–	–
	KU mit depressiver Stimmung	42^c^	1,07	90,5	–	–	–
	KU mit positiven Emotionen	139	21,50**	100	T2/T1	1,41	0,12
					T1/T3	−3,03*	0,26
					T2/T3	−4,44**	0,38
*EKF*	*Gesamt*	174	0,09	100	–	–	–
	Erkennen eigener Emotionen	173	4,81	100	–	–	–
	Erkennen von Emotionen anderer	174	0,49	100	–	–	–
	Regulation von Emotionen	174	2,89	100	–	–	–
	Emotionale Expressivität	174	1,64	100	–	–	–

Die Gesamtskalen sind *kursiv* hervorgehoben

*EKF* Emotionale-Kompetenz-Fragebogen, *SEK-ES* Fragebogen zur emotionsspezifischen Selbsteinschätzung emotionaler Kompetenzen, *H* erlebte Häufigkeit, *KU* konstruktiver Umgang, *n* Anzahl der Personen, zu denen Daten zu allen 3 Messzeitpunkten vorlagen, *r* Effektstärkemaß, *r* *>* 0,10 kleiner, *r* *>* 0,30 mittlerer, *r* *>* 0,50 starker Effekt [24]

*Signifikant bei einem *α* von 2,5 %/*p* *<* 0,025

**Signifikant bei einem *α* von 0,1 %/*p* *<* 0,001

^a^Power der Tests zur Detektion eines klinisch relevanten mittelgroßen Effekts (*f* = 0,25), Grundlage: Spearman-Korrelation

^b^Signifikanzwerte wurden mittels Bonferroni-Korrektur für mehrere Tests angepasst

^c^Die geringe Personenanzahl ergab sich aus der Instruktion des Fragebogens. Die Items zum Umgang mit einzelnen Emotionen durften laut Fragebogeninstruktion nur beantwortet werden, wenn die jeweilige Emotion in der letzten Woche wahrgenommen wurde

**Abb. 1:**
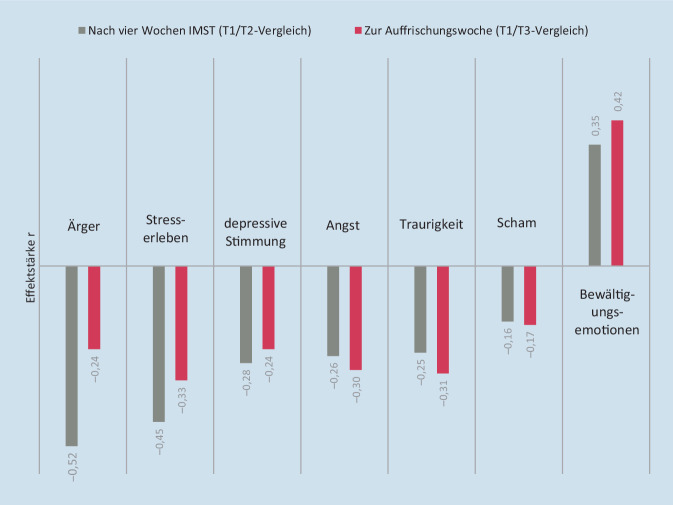
Effektstärke im Verlauf der Therapie zur Illustration der signifikant veränderten Häufigkeitsangaben konkreter Emotionen (SEK-ES). *SEK-ES* Fragebogen zur emotionsspezifischen Selbsteinschätzung emotionaler Kompetenzen, *T1* Therapiebeginn, *T2* Therapieende, *T3* Auffrischungswoche, 14 Wochen nach Therapiebeginn, *r* Effektstärkemaß: *r* *>* 0,10 kleiner, *r* *>* 0,30 mittlerer, *r* *>* 0,50 starker Effekt [24]. ^a^Für eine erleichterte Interpretierbarkeit stehen im Diagramm positive Effektstärken für eine Zunahme im Verlauf der Therapie und negative Effektstärken für eine Reduktion im Verlauf der Therapie

Die größte Veränderung wurde beim Erleben von *Ärger* (T2/T1: *z* = 6,91; *p* < 0,001; *r* = 0,52, Tab. 2; Abb. 1) sichtbar. Die untersuchten Personen hatten zum Therapieende bedeutend seltener *Ärger*. Eine mittelgroße Reduktion konnte weiterhin für die Häufigkeit des *Stresserlebens* nachgewiesen werden (T2/T1: *z* = 6,00; *p* < 0,001; *r* = 0,45). Bewältigungsemotionen wurden im mittelstarken Ausmaß häufiger berichtet (T1/T3: z = −5,52; *r* = 0,42; Tab. 2; Abb. 1).

Auch hinsichtlich des *Angsterlebens* (SEK-ES: T3/T1: *z* = 3,95; *p* < 0,001; *r* = 0,30) und der Häufigkeit von *Traurigkeit* (T3/T1: *z* = 4,08; *p* < 0,001; *r* = 0,31) konnte eine mittelstarke Verringerung zu T3 festgestellt werden (Tab. 1 und 2; Abb. 1).

Zudem war eine kleine Verringerung der *depressiven Stimmung* zu T2 feststellbar (T2/T1:* z* = 3,68; *p* = 0,001; *r* = 0,28; Tab. 2).

Das leicht reduzierte Schamerleben war in den vergleichenden Post-hoc-Tests nicht mehr statistisch gegen den Zufall abgesichert (T3/T1: *z* = 2,29; *r* *=* 0,17; *p* = 0,067; Tab. 1 und 2). Für die Emotionen *Ekel* (*χ*^2^ (2) = 4,99; *p* *=* 0,082) und *Schuld* (*χ*^2^ (2) = 7,09; *p* *=* 0,026) waren bei einem *α* von 2,5 % keine statistisch signifikanten Veränderungen nachweisbar (Tab. 1 und 2).

### Veränderung der emotionalen Kompetenz

Auf Gesamt- und Subskalenebene blieb die Selbsteinschätzung mittels EKF konstant (z. B. Gesamtskala des EKF: *M*_T1_ = 99,58; *SD*_T1_ = 7,92; *M*_T2_ = 99,30; *SD*_T2_ = 7,78; *M*_T3_ = 99,65, *SD*_T3_ = 7,99; *χ*^2^_EKF__*_gesamt*_ (2) = 0,09; *p* *=* 0,956; Tab. 1 und 2). Zu allen drei Messzeitpunkten erlebten sich die Befragten als durchschnittlich emotional kompetent im Vergleich zur Normstichprobe. Auch im SEK-ES-Fragebogen waren keine Veränderungen im Hinblick auf den selbst beurteilten generellen und spezifischen *konstruktiven Umgang mit Emotionen* eruierbar (z. B. Gesamtskala des SEK_ES *χ*^2^_*konstruktiver_Umgang*_ (2) = 2,63; *p* *=* 0,269; Tab. 1 und 2). Einzige Ausnahme bildete die selbst eingeschätzte Kompetenz zum *konstruktiven Umgang mit positiven Emotionen*. Diese war zum Therapieende zunächst in kleinem statistisch nicht signifikantem Ausmaß weniger, nahm bis zur Auffrischungswoche jedoch mittelstark zu (T2/T1: *z* = 1,41; *p* *=* 0,476; *r* = 0,12; T2/T3: *z* = −4,44; *p* < 0,001; *r* = 0,38).

### Vorhersage der Häufigkeit von Emotionen durch emotionale Kompetenz

46,8 % der Varianz der wahrgenommenen *Häufigkeit positiver Emotionen* und 39 % der Varianz der berichteten *Häufigkeit negativer Emotionen* konnte durch den *konstruktiven und kompetenten Umgang mit Emotionen* (SEK-ES) erklärt werden (Tab. 3).

**Tab. 3:** Einfache lineare Regression der Häufigkeit positiver und negativer Emotionen (SEK-ES, Kriterium) mittels der Kompetenzen im konstruktiven Umgang mit Emotionen (SEK-ES, Prädiktor)^a^

Kriterium (T3)	Prädiktor (T3) emotionale Kompetenz	*n*	*B*	*β*	Konstante	Korr. *R*^2^	*F*	Power^b^ in %
Häufigkeit positiver Emotionen	Konstruktiver Umgang mit Emotionen (gesamt)	163	0,84**	0,69	0,29	0,468	144,34**	100
Häufigkeit negativer Emotionen	Konstruktiver Umgang mit Emotionen (gesamt)	164	−0,68**	−0,63	2,86	0,390	105,77**	100

*SEK-ES* Fragebogen zur emotionsspezifischen Selbsteinschätzung emotionaler Kompetenzen, *T3* Auffrischungswoche, *n* Personenanzahl, *B* unstandardisierter Regressionskoeffizient, *β* standardisierter Regressionskoeffizient, *F* Wert der Varianzanalyse zur Überprüfung der Signifikanz des Regressionsmodells, *R*^2^ Determinationskoeffizient, erklärte Varianz, Parameter zur Einschätzung der Modellgüte und Effektstärke, *R*^2^ = 0,02 schwacher Effekt, *R*^2^ = 0,13 mittlerer Effekt und *R*^2^ = 0,26 großer Effekt nach Cohen [24]

**p *<* 0,001

^a^Berechnungen wurden mittels Bootstrapping-Verfahren aufgrund nicht vollständig erfüllter statistischer Voraussetzungen bestätigt

^b^Empirische Teststärke

## Diskussion

Die vorliegende Studie konnte klinisch relevante Veränderungen der von den Patient*innen berichteten *Häufigkeiten positiv und negativ erlebter Emotionen* während der tagesklinischen IMST nachweisen. Positive Emotionen wurden nach der Therapie häufiger und negative Emotionen seltener berichtet. In Anbetracht der Bedeutsamkeit von Emotionen für das Schmerzerleben [8] kann dieser Befund als potenzieller Erfolgsmarker der IMST eingeordnet werden. Vorangegangene Untersuchungen zu Veränderungen von *Angst* und *Depressivität* während einer IMST wurden in dieser Studie repliziert [17, 18]. Auch in der vorliegenden Forschungsarbeit konnte diesbezüglich eine Reduktion eruiert werden. Im Besonderen imponierte die starke Reduktion der Emotion *Ärger* zum Therapieende. Dies lässt den Schluss zu, dass sich der zugrunde liegende Umgang mit Ärger während der IMST änderte. Jüngste Forschung unterstreicht die besondere Bedeutung von Ärger und Ärgermanagement bei chronifiziertem Schmerz [25–28], so beispielsweise bei Migräne [29]. Überblicksarbeiten definieren kleine bis mittlere Zusammenhänge zwischen ärgerassoziierten Variablen und Schmerzintensität bzw. schmerzassoziierten Beeinträchtigungen [26]. Weiterhin kam es in der vorliegenden Verlaufsmessung zu einer Zunahme von *Bewältigungsemotionen*. Diese Unterkategorie der positiven Emotionen umfasst Emotionen wie Mut, Zuversicht oder Dankbarkeit und unterstützt Personen im konstruktiven Meistern von situativen Herausforderungen. Frühere Forschung konnte bereits nachweisen, dass positive Emotionen im Allgemeinen und Optimismus im Konkreten bei Menschen mit chronischen muskuloskeletalen Schmerzen zur Ausdauer bei der Erledigung von Aufgaben, einem (schmerz‑)flexiblen Zielmanagement und einem verringerten Vermeidungsverhalten beitragen [30]. Mit der vorliegenden Studie vergleichbare bewältigungsemotions- und ärgerspezifische *Verlauf*suntersuchungen bei Menschen mit chronifizierten Schmerzen im interdisziplinären tagesklinischen Setting liegen bisher nicht vor.

Das emotionale *Kompetenzerleben* der Patient*innen blieb während der Therapie – im Gegensatz zur erlebten Häufigkeit bestimmter Emotionen – konstant. Vor dem Hintergrund der emotionsfokussierten psychotherapeutischen Techniken im Rahmen der Gruppenpsychotherapie irritiert zunächst die fehlende Verbesserung der selbst wahrgenommenen emotionalen Kompetenzen, z. B. hinsichtlich des Erkennens von Emotionen. Eine Erklärung könnte einerseits sein, dass die Interventionen die *Einschätzung* emotionaler Fertigkeiten und das emotionale Selbstwirksamkeitserleben der Patient*innen bisher unzureichend adressierten. Andererseits erlebten sich die untersuchten Personen im Vergleich zur Normstichprobe/Normalbevölkerung bereits von Therapiebeginn an als *durchschnittlich* emotional kompetent, sodass es nachvollziehbar ist, wenn mögliche Verbesserungen des eigenen kompetenten Umgangs für die Befragten wenig salient waren. Dennoch konnte in der Untersuchung ein hoher prädiktiver Wert emotionaler Kompetenzen für die Emotionshäufigkeit nachgewiesen werden. Vor diesem Hintergrund stellt sich die Frage, ob sich angesichts der starken Veränderungen der Häufigkeiten positiver und negativer Emotionen die EK implizit verändert hat, ohne dass die Patient*innen dies als *Kompetenzverbesserung* beurteilten. Offen bleibt, inwiefern das Selbstbeurteilungsinstrument ausreichend geeignet war einen möglichen Kompetenzzuwachs zu detektieren. Interviews, Videoanalysen oder physiologische Parameter sollten für zukünftige Forschung als alternative Messverfahren berücksichtigt werden. Gleichzeitig lohnen sich weiterführende Überlegungen, welche konkreten Ergebniskriterien die schmerz-/alltagsrelevanten Auswirkungen einer potenziell veränderten emotionalen Kompetenz bestmöglich abbilden.

Limitierend anzumerken ist, dass das deskriptive Format der vorliegenden Studie die Generalisierbarkeit der Ergebnisse einschränkt. Aufgrund der fehlenden Kontrollgruppe kann keine kausale Verbindung zwischen IMST und der Verbesserung des emotionalen Erlebens hergestellt werden. Es wird lediglich eine Veränderung während der IMST beschreibend festgehalten. Da Emotionen per se instabil sind [1], können die emotionalen Veränderungen auch unabhängig von möglichen Therapieeffekten entstanden sein. Dies gilt es nun weiterführend zu überprüfen. Auch die unzureichende scharfe Abgrenzung der Konzepte *Gefühle, Emotion, Stimmung *im Erhebungsinstrument SEK-ES kann als kritischer Aspekt gewertet werden: Eine depressive Stimmung beispielsweise ist, im engeren Sinne, keine Emotion. Dies sollte bei der Interpretation der Ergebnisse berücksichtigt werden. Weiterhin kritisch anzumerken ist die ungleich differenzierte Erfassung positiv und negativ konnotierter Emotionen: Während im SEK-ES zu den negativ assoziierten Emotionen emotions*spezifische* Aussagen getroffen werden können, können im Bereich der positiv aufgeladenen Emotionen lediglich zur Subkategorie der Bewältigungsemotionen differenzierte Aussagen getätigt werden.

Zusammenfassend lässt sich festhalten, dass die vorliegende explorative Verlaufsmessung eine deutliche Zunahme positiv erlebter Emotionen und eine Abnahme negativ erlebter Emotionen während der IMST nachweisen konnte. Trotz des unveränderten emotionalen *Kompetenzempfindens* legt das klinisch relevant veränderte *emotionale Erleben* der Patient*innen die Schlussfolgerung nahe, dass während der IMST implizit und explizit emotionsrelevante Veränderungen stattfinden. Spannend bleibt, welche Aspekte der Therapie genau potenziell zum veränderten emotionalen Erleben beitragen. Weitere Untersuchungen zum verbesserten Verständnis darunterliegender Mechanismen bieten sich an. Interessant ist hierbei, ob Subgruppen sich hinsichtlich ihrer EK und ihres emotionalen Erlebens unterschiedlich stark verändern oder von spezifischen EK-Interventionen unterschiedlich intensiv profitieren. Weitere vertiefende Forschungsperspektiven könnten zudem sein, die eruierte Veränderung des emotionalen Erlebens während der IMST auf geschlechterspezifische oder altersbezogene Unterschiede differenzierter zu untersuchen. Auch sollten die gefundenen Evidenzen hinsichtlich möglicher Interaktionen mit anderen schmerzrelevanten Aspekten, wie Schmerzakzeptanz und psychischer Flexibilität, genauer beleuchtet werden.

Generell empfiehlt sich für zukünftige Behandlungskonzeptionen und -evaluierungen, die Aufmerksamkeit mehr auf Veränderungen des emotionalen Erlebens und der dahinterstehenden emotionalen Kompetenzen der Patient*innen zu richten. Forschungen, die verstärkt emotionsfokussierende Interventionen in die Schmerztherapie einbinden [13, 14], verzeichneten bereits inkrementelle schmerzassoziierte Verbesserungen. Die in dieser Studie explorierten Evidenzen zum veränderten emotionalen Erleben bieten Anregung für zukünftige innovative Blickwinkel auf das bereits erfolgreiche Therapiekonzept IMST.

## References

[1] Barnow S (2020) Konzepte und Modelle von Emotion und Emotionsregulation. In: Barnow S (Hrsg) Handbuch Emotionsregulation: Zwischen psychischer Gesundheit und Psychopathologie. Springer, Berlin, Heidelberg, S 3–18

[2] Barrett LF (2017) The theory of constructed emotion: an active inference account of interoception and categorization. Soc Cogn Affect Neurosci 12:1–23 27798257 10.1093/scan/nsw154 PMC5390700

[3] Wormwood JB, Quigley KS, Barrett LF (2022) Emotion and threat detection: the roles of affect and conceptual knowledge. Emotion 22:1929–1941 34081492 10.1037/emo0000884

[4] Bennett D, Davidson G, Niv Y (2022) A model of mood as integrated advantage. Psychol Rev 129:513–541 34516150 10.1037/rev0000294 PMC8917968

[5] Rindermann H (2009) Emotionale-Kompetenz-Fragebogen (EKF). Hogrefe, Göttingen

[6] Berking M (2015) Training emotionaler Kompetenzen. Springer, Berlin

[7] International Association for the Study of Pain (IASP) IASP announces revised definition of pain. https://www.iasp-pain.org/publications/iasp-news/iasp-announces-revised-definition-of-pain/. Zugegriffen: 19. Dez. 2022

[8] Wiech K, Tracey I (2009) The influence of negative emotions on pain: behavioral effects and neural mechanisms. Neuroimage 47:987–994 19481610 10.1016/j.neuroimage.2009.05.059

[9] Konietzny K, Suchan B, Kreddig N et al (2016) Emotionsregulation und Schmerzen. Behaviorale und neuronale Korrelate – ein transdiagnostischer Ansatz. Schmerz 30:412–420 27658393 10.1007/s00482-016-0162-1

[10] Scheer C, Kubowitsch S, Dendorfer S et al (2021) Happy enough to relax? How positive and negative emotions activate different muscular regions in the back—An explorative study. Front Psychol 12:511746 34135791 10.3389/fpsyg.2021.511746 PMC8201496

[11] Lumley MA, Cohen JL, Borszcz GS et al (2011) Pain and emotion: a biopsychosocial review of recent research. J Clin Psychol 687:942–968 10.1002/jclp.20816 PMC3152687 21647882

[12] Tefft AJ, Jordan IO (2016) Eye movement desensitization reprocessing as treatment for chronic pain syndromes: a literature review. J Am Psychiatr Nurses Assoc 22:192–214 27048429 10.1177/1078390316642519

[13] Yarns BC, Lumley MA, Cassidy JT et al (2020) Emotional awareness and expression therapy achieves greater pain reduction than cognitive behavioral therapy in older adults with chronic musculoskeletal pain: a preliminary randomized comparison trial. Pain Med 21:2811–2822 32451528 10.1093/pm/pnaa145

[14] Lumley M, Schubiner H (2019) Emotional awareness and expression therapy for chronic pain: rationale, principles and techniques, evidence, and critical review. Curr Rheumatol Rep 21:1–8 31123837 10.1007/s11926-019-0829-6 PMC7309024

[15] Lumley MA, Krohner S, Marshall LM et al (2021) Emotional awareness and other emotional processes: implications for the assessment and treatment of chronic pain. Pain Manag 11:325–332 33533272 10.2217/pmt-2020-0081 PMC7923252

[16] Arnold B, Brinkschmidt T, Casser H‑R et al (2009) Multimodale Schmerztherapie. Konzepte und Indikation. Schmerz 23:112–120 19156448 10.1007/s00482-008-0741-x

[17] Schütze A, Kaiser U, Ettrich U et al (2009) Evaluation einer multimodalen Schmerztherapie am UniversitätsSchmerzCentrum Dresden. Schmerz 23:609–617 19756770 10.1007/s00482-009-0827-0

[18] Zhuk A, Schiltenwolf M, Neubauer E (2018) Langfristige Wirksamkeit einer multimodalen Schmerztherapie bei chronischen Rückenschmerzen. Nervenarzt 89:546–551 28831509 10.1007/s00115-017-0391-2

[19] Deutsche Schmerzgesellschaft e. V. Deutscher Schmerzfragebogen. https://www.schmerzgesellschaft.de/schmerzfragebogen. Zugegriffen: 27. Jan. 2023

[20] Pardos-Gascón EM, Narambuena L, Leal-Costa C et al (2021) Differential efficacy between cognitive-behavioral therapy and mindfulness-based therapies for chronic pain: systematic review. Int J Clin Health Psychol 21:100197 33363580 10.1016/j.ijchp.2020.08.001 PMC7753033

[21] Ebert D, Christ O, Berking M (2012) Fragebogen zur emotionsspezifischen Selbsteinschätzung emotionaler Kompetenzen. Open Testarchiv Trier, Trier

[22] Körner AJ, Sabatowski R, Burdic L et al (2023) Emotionale Kompetenzen bei Menschen mit chronifizierten Schmerzen: ein Selbst und Fremdbild. Schmerz. 10.1007/s00482-023-00720-x 37278837 10.1007/s00482-023-00720-x PMC10959775

[23] Sabatowski R, Kaiser U, Scharnagel R (2021) Interdisziplinäre multimodale Schmerztherapie – Grundlagen und Fallstricke. Anasth Intensivmed 62:334–344

[24] Cohen J (1992) A power primer. Psychol Bull 112:155–159 19565683 10.1037//0033-2909.112.1.155

[25] Yarns BC, Cassidy JT, Jimenez AM (2022) At the intersection of anger, chronic pain, and the brain: a mini-review. Neurosci Biobehav Rev 135:104558 35122780 10.1016/j.neubiorev.2022.104558

[26] Adachi T, Yamada K, Fujino H et al (2022) Associations between anger and chronic primary pain: a systematic review and meta-analysis. Scand J Pain 22:1–13 34908255 10.1515/sjpain-2021-0154

[27] Galvez-Sánchez CM, Reyes del Paso GA, Duschek S et al (2022) The link between fibromyalgia syndrome and anger: a systematic review revealing research gaps. J Clin Med 11:844 35160295 10.3390/jcm11030844 PMC8836473

[28] Yamada K, Fujii T, Kubota Y et al (2022) Negative effect of anger on chronic pain intensity is modified by multiple mood states other than anger: a large population-based cross-sectional study in Japan. Mod Rheumatol 32:650–657 34910207 10.1093/mr/roab035

[29] Shaygan M, Saranjam E, Faraghi A et al (2022) Migraine headaches: the predictive role of anger and emotional intelligence. Int J Community Based Nurs Midwifery 10:74–83 35005043 10.30476/IJCBNM.2021.90552.1706 PMC8724728

[30] Esteve R, López-Martínez AE, Peters ML et al (2018) Optimism, positive and negative affect, and goal adjustment strategies: their relationship to activity patterns in patients with chronic musculoskeletal pain. Pain Res Manag 2018:1–12 10.1155/2018/6291719 PMC5875047 29736198

